# A Novel Contrast-Induced Acute Kidney Injury Model Based on the 5/6-Nephrectomy Rat and Nephrotoxicological Evaluation of Iohexol and Iodixanol *In Vivo*

**DOI:** 10.1155/2014/427560

**Published:** 2014-11-11

**Authors:** Tong-qiang Liu, Wei-li Luo, Xiao Tan, Yi Fang, Jing Chen, Hui Zhang, Xiao-fang Yu, Jie-ru Cai, Xiao-qiang Ding

**Affiliations:** ^1^Division of Nephrology, Zhongshan Hospital, Fudan University, Shanghai 200032, China; ^2^Division of Nephrology, The Affiliated Changzhou No. 2 Hospital of Nanjing Medical College, Changzhou, Jiangsu 213003, China; ^3^Shanghai Institute of Kidney and Dialysis, Shanghai 200032, China

## Abstract

Contrast-induced acute kidney injury (CI-AKI) is a serious complication in patients after administration of iodinated contrast media. Proper animal models of CI-AKI can help understand the mechanisms involved and prevent the disorder. We used the 5/6-nephrectomized (NE) rat to develop a CI-AKI model and to evaluate differences in the toxic effects on the kidney between iohexol and iodixanol. We found that six weeks after ablative surgery was the preferred time to induce CI-AKI. We compared multiple pretreatment plans and found that dehydration for 48 hours before iodixanol (320, 10 mL/kg) administration was optimal to induce CI-AKI in the 5/6 NE rats. Compared with iodixanol, iohexol induced a significantly greater reduction in renal function, severe renal tissue damage, intrarenal hypoxia, and apoptotic tubular cells. Iohexol and iodixanol resulted in similarly marked increases in levels of inflammation and oxidative stress. In summary, the 5/6 NE rat combined with dehydration for 48 hours is a useful pretreatment to establish a novel and reliable CI-AKI model. Iohexol induced more severe CI-AKI than iodixanol in this model.

## 1. Introduction

Recent advances in medical technology have led to an increased use of iodinated contrast media (ICM) in radiographic diagnostic and interventional procedures [1]. However, ICM has a toxic effect on renal tubules [2]. Contrast-induced acute kidney injury (CI-AKI) is now the third most common cause of hospital-acquired renal failure. CI-AKI is associated with increased costs of medical care and long admissions and is a strong predictor of poor early and late outcomes [1, 3], especially in patients who are in need of dialysis [4]. Therefore there is a clear need to establish an appropriate and reproducible experimental animal model that can be used to help understand and prevent this disease. Clinical data have shown a significantly lower frequency of CI-AKI in patients with impaired renal function who received low-osmolar contrast medium (LOCM) or isoosmolar contrast medium (IOCM), leading to the use of LOCM and IOCM in clinical practice instead of high-osmolar contrast medium (HOCM) [1, 3]. Unfortunately, previous CI-AKI models are not appropriate since HOCM was administered [5–9] and/or prepared with pharmacological procedures [2, 6, 9–11]. The drug makes studies involving an interaction of ICM with pharmacologic agents hazardous. Moreover, there is no animal model available to study IOCM-induced AKI. The need for a reliable animal model to study LOCM- and IOCM-induced AKI is crucial. Chronic kidney disease (CKD) is considered the most important risk factor for CI-AKI in humans [1, 3]. Several epidemiologic studies have indicated that the prevalence of CKD was 10.8~16.8% in the general population and had shown an increasing trend [12, 13]. It is indispensable to study CI-AKI based on CKD. However, a LOCM- and IOCM-induced AKI animal model based on CKD can be reliably utilized has challenged the field research. Iodixanol is an IOCM and iohexol is an LOCM. The question of whether iodixanol is superior to iohexol remains controversial.

In the present study, the main objective was to establish a new, highly reliable and reproducible model of CI-AKI. An additional aim was to compare the toxic effects of iohexol and iodixanol on the kidney using the new model.

## 2. Materials and Methods

### 2.1. Chemicals and Animals

The nonionic ICM were (i) the LOCM, iohexol (350 mg iodine/mL, 844 mOsm/kg of water and 10.4 cPs at 37°C; 300 mg iodine/mL, and 672 mOsm/kg of water and 6.3 cPs at 37°C; GE Healthcare, Shanghai, China) and (ii) an IOCM, iodixanol (320 mg iodine/mL, 290 mOsm/kg of water and 11.8 cPs at 37°C (GE Healthcare)). Male Sprague-Dawley rats (180–200 g) were purchased from the Animal Center of Fudan University, Shanghai, China. The rats were acclimatized for 7 d before the start of study and handled in accordance with the institutional and national guidelines for animal research. The 5/6 NE was performed under a 4% sodium pentobarbital (40 mg/kg) intraperitoneal anesthesia by a nephrectomy of the right kidney and resection of two thirds of the left kidney, as described previously [14, 15]. All experimental protocols were approved by the Animal Care and Use Committee of Fudan University and in compliance with Guidelines for the Care and Use of Laboratory Animals published by the National Academy Press (NIH Publication number 85-23, revised 1996).

### 2.2. *In Vivo* Experimental Design

**Figure 1:**
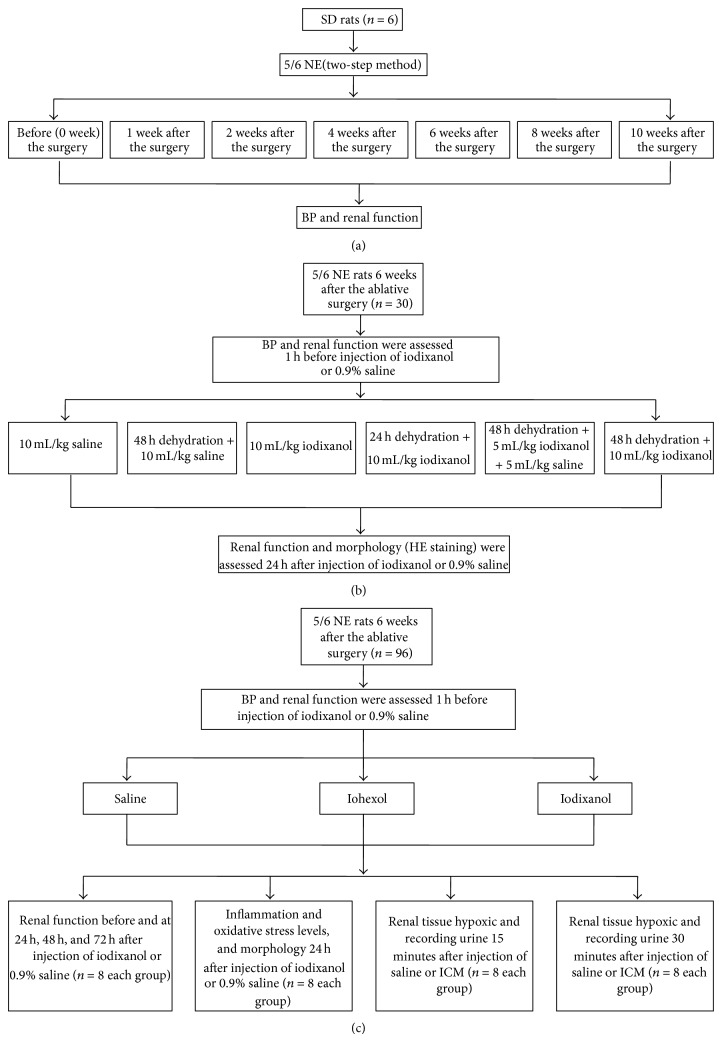
Study protocol.

The study was divided into three phases. The first phase involved a change trend of renal function study in the 5/6 NE rats to find an appropriate time to establish the CI-AKI model (Figure 1(a)): six rats were enrolled to observe dynamic changes in renal function. Urine and blood samples were taken before (week 0) and at 1, 2, 4, 6, 8, and 10 weeks after the ablative surgery. Serum and urine creatinine in 5/6 NE rats were also measured before (week 0) and at 1, 2, 4, 6, 8, and 10 weeks after the ablative surgery. In this study, there was a relatively stable period of renal function from 4 to 8 weeks after the ablative surgery. Renal function was most stable 6 weeks after the ablative surgery. Six weeks after the surgery was therefore chosen as the preferred time for phases two and three. The second phase involved multiple dehydration pretreatment plans to find the optimal precondition procedure (Figure 1(b)). Thirty rats with similar renal function 6 weeks after the ablative surgery were enrolled and randomly assigned to six experimental groups (*n* = 5 each): (1) saline group: saline was injected at 10 mL/kg via tail vein administration; (2) 48 h dehydration + saline group: after dehydration for 48 hours, 0.9% saline was injected at 10 mL/kg via tail vein administration; (3) iodixanol group: iodixanol (320) was injected at 10 mL/kg via tail vein administration; (4) 24 h dehydration + iodixanol group: after dehydration for 24 hours, iodixanol was injected at 10 mL/kg via tail vein administration; (5) 48 h dehydration + low dose iodixanol group: after dehydration for 48 hours, 5 mL/kg iodixanol and 5 mL/kg saline were mixed and injected via tail vein administration, and (6) 48 h dehydration + iodixanol group: after dehydration for 48 hours, iodixanol was injected at 10 mL/kg via tail vein administration. BP was measured 1 h before administration of contrast media or 0.9% saline. We found combination dehydration for 48 hours with iodixanol (10 mL/kg) administration resulted in CI-AKI. The third phase involved studying differences in the toxic effects on the kidney between iohexol and iodixanol using the new model (Figure 1(c)): ninety-six rats with similar renal function 6 weeks after the ablative surgery were enrolled and randomly assigned to three experimental groups (*n* = 32 each). All rats were dehydrated for 48 hours before injection of ICM (3.2 g I/kg) and/or 0.9% saline via tail vein administration. (1) 0.9% saline was injected at 10 mL/kg, control group; (2) iohexol (350) 4 mL/kg and iohexol (300) 6 mL/kg were mixed to match the iodine concentration of iodixanol (320) and injected, iohexol group; (3) iodixanol was injected at 10 mL/kg, iodixanol group. Each experimental group was then divided randomly into four equal subgroups (*n* = 8 in each): two subgroups for assessment of renal tissue hypoxic and recording urine volume by bladder catheterization (15 min and 30 min after injection of saline or ICM) and other two subgroups for renal function, inflammation and oxidative stress levels, and morphology respectively. Animals were given saline, iohexol, or iodixanol intravenously at a rate of 1 mL/kg*·*bw/min. All animals had ad libitum access to water and food after injection. The animals were again anesthetized with sodium pentobarbital and the kidney was cut sagittally 15 min, 30 min, or 24 h after the injection. Systolic blood pressure (BP) was assessed by tail cuff. Urine samples were collected 24 h in metabolism cages placed on a urine collection refrigerated rack. Approximately 1 mL of blood was taken from the jugular vein in the untreated rats and from the abdominal aorta in killed rats. Blood was allowed to clot for a minimum of 45 min, and serum was collected after centrifugation at 2000 g for 10 min.

### 2.3. Rat Serum and Urine Biomarkers Were Investigated in Three Phases

Serum creatinine (Scr) and blood urea nitrogen (BUN) concentrations were determined using a Hitachi 7060 chemistry analyzer. Creatinine clearance (Ccr) was calculated as mL/min/kg. Serum TNF-*α* levels were determined using commercial enzyme-linked immunosorbent assay (ELISA) kits (R&D Systems, Inc., Minneapolis, MN, USA) according to the manufacturer's instructions.

### 2.4. Haematoxylin and Eosin Staining

Kidney tissue was fixed in 10% neutral-buffered formalin for a minimum of 24 hours, embedded in paraffin, and tissue sections 3 *μ*m thick were cut using a microtome and stained with hematoxylin-eosin for histopathological evaluation. Stained specimens were assessed by a pathologist in a blinded fashion using a light microscope (Leica DM 6000 B; Leica Microsystems, Wetzlar, Germany). For semiquantitative analysis of the frequency and severity of renal lesions, we selected randomly 10 high-magnification (×200) fields of the cortex and outer stripe of the outer medulla. The specimens were scored according to the extent of foamy degeneration and detachment of tubular cells on a semiquantitative scale [16]: no injury (0), mild: <25% (1), moderate: <50% (2), severe: <75% (3), and very severe: >75% (4).

### 2.5. Immunohistochemistry Staining for Inflammation Markers (ED-1 and TNF-*α*)

Immunohistochemistry staining was performed in 3 *µ*m paraffinized sections. The samples were dewaxed and dehydrated, washed in phosphate-buffered saline (PBS), and incubated with 3% H_2_O_2_ for 10 min to eliminate endogenous peroxidase activity and then treated with normal goat serum (1 : 20) for 20 min. Next, incubation with anti-ED-1 antibody (rabbit monoclonal, 1 : 200; Abcam, Cambridge, MA, USA) or TNF-*α* (rabbit polyclonal, 1 : 2000; Abcam) was performed at 4°C overnight. The sections were then incubated with horseradish peroxidase-conjugated secondary antibody (anti-rabbit IgG). After rinsing in PBS three times, the sections were stained with 3, 3′-diaminobenzidine (Sigma, Shanghai, China) and then counterstained with hematoxylin and evaluated under a light microscope. Stained specimens were assessed by a pathologist in a blinded fashion. We selected randomly five high-magnification (×200) fields of the renal corticomedullary boundary zone. The specimens were scored according to the percentage of ED-1-positive cells and the extent and intensity of TNF-*α*.

### 2.6. Immunofluorescent Labeling for Theoxidized Derivative of Deoxyguanosine (8-OHdG)

Immunofluorescent labeling was performed on the frozen sections of the renal corticomedullary boundary zone. After fixation in acetone for 10 min, the sections were treated with normal goat serum for 20 min. Subsequently, they were incubated at 4°C overnight with anti-8-OHdG antibody (mouse monoclonal, 1 : 500; Abcam). Then the sections were incubated with goat anti-mouse antibodies conjugated with Alexa Fluor 488 (1 : 100; Abcam), before the nuclei were counterstained with 4′, 6-diamidino-2-phenylindole (DAPI, 2 mg/mL). The slides were then examined under a fluorescence microscope (Leica TCS SP5). Fluorescence intensity was measured using a ×63 objective microscope in five different views per section.

### 2.7. Lipid Peroxidation/ROS Production

MDA is a naturally occurring product of lipid peroxidation and an indicator of ROS production. Supernatant of the renal cortical homogenate was determined according to the manufacturer's protocol (TBARS Assay Kit; Cayman Chemical Company, Ann Arbor, Michigan, USA). The level of lipid peroxides was expressed as nmol of MDA/g of kidney.

### 2.8. *In Vivo* Labeling of Hypoxic

Renal tissue hypoxia was assessed at 15 min or 30 min after an intravenous injection of saline, iohexol, or iodixanol using the Hypoxyprobe-1 Omni Kit (Natural Pharmacia International Inc., Burlington, MA, USA), which contains pimonidazole hydrochloride and rabbit polyclonal anti-pimonidazole. Pimonidazole remains in hypoxic cells after forming an irreversible adduct with thiol groups in environments with PO_2_  <10 mmHg. These protein adducts are effective immunogens for rabbit anti-pimonidazole antisera. At 60 min prior to being killed, each rat received injections via the tail vein of Hypoxyprobe-1 in a 0.5 mL bolus (60 mg/kg*·*bw). Immunohistochemical staining was performed on the renal sections of the renal medulla. Paraffinized sections (3 *µ*m) were dewaxed and dehydrated, washed in PBS, and incubated with 3% H_2_O_2_ for 5 min to quench tissue peroxidase activity and were then treated with normal goat serum (1 : 20) for 5 min. Afterwards, they were incubated with rabbit polyclonal anti-pimonidazole (1 : 500) for 40 min. The sections were then incubated with horseradish peroxidase-conjugated secondary antibody (anti-rabbit IgG). After rinsing in PBS three times, the sections were stained with 3, 3′-diaminobenzidine (Sigma) and then counterstained with hematoxylin and evaluated under a light microscope. Five high-magnification (×200) fields of the outer medulla were selected randomly and assessed by a pathologist in a blinded fashion. The specimens were scored by the extent and intensity of Hypoxyprobe.

### 2.9. dUTP Nick-End Labeling Assay

To determine renal tubular cells apoptosis, terminal deoxynucleotidyl transferase-mediated dUTP nick-end labeling (TUNEL) assay was performed on the frozen sections of the renal corticomedullary boundary zone with a commercial kit (In situ Cell Death Detection kit; Roche, Basel, Switzerland) according to the manufacturer's protocol. The samples were fixed in acetone for 10 min, washed in PBS, and immersed in a solution of 3% H_2_O_2_ to eliminate endogenous peroxidase activity. Incubation with the TUNEL reaction mixture was then performed for 60 min. The number of TUNEL-positive cells and total cell number in kidney sections were counted under a fluorescence microscope (Leica TCS SP5), before the nuclei were counterstained with 2 mg/mL DAPI. All cells were counted using a ×63 objective microscope in five different views per section. TUNEL-positive cells were expressed as percentage of total cells.

### 2.10. Statistical Analysis

Statistical analysis was performed using the statistical software SPSS Version 16.0. All data are presented as means ± SD. The means for groups were evaluated by analysis of variance followed by Tukey's multiple comparison. A *P* value of <0.05 was considered significant.

## 3. Results

### 3.1. BP Increased Gradually after 5/6 NE in the Rats and Renal Function Was Most Stable 6 Weeks after the Ablative Surgery

**Figure 2:**
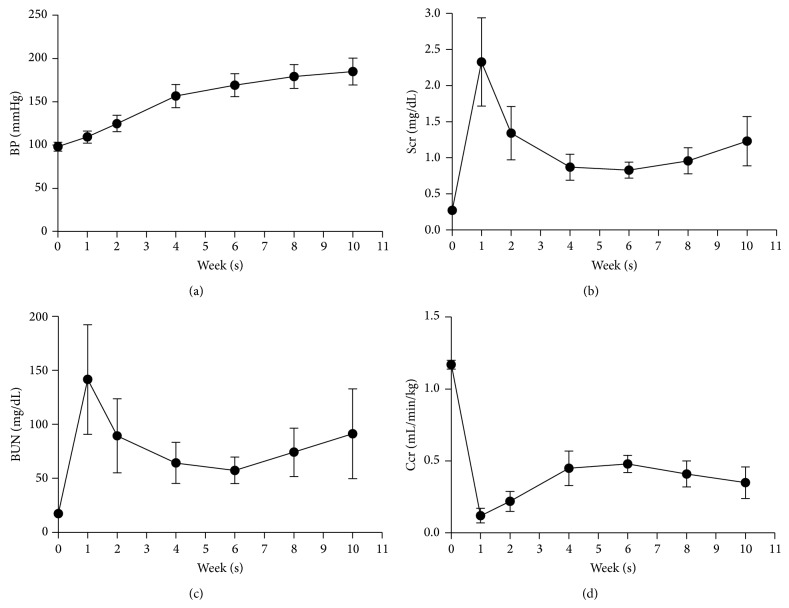
BP increased gradually after 5/6 NE in rats and renal function was most stable 6 weeks after the ablative surgery. Changes in the levels of (a) BP, (b) Scr, (c) BUN, and (d) Ccr before (week 0) after (1, 2, 4, 6, 8, and 10 weeks) 5/6 NE in rats; *n* = 6.

BP increased gradually after 5/6 NE in the rats and was 168.8 ± 13.1 mmHg 6 weeks after the ablative surgery (Figure 2(a)). Rats showed a marked reduction in renal function after the ablative surgery. Figures 2(b)–2(d) show the change in renal function before and after the ablative surgery. Renal function reached a definite plateau 4–8 weeks after the surgery. Renal function was most stable 6 weeks after the ablative surgery (Scr, 0.83 ± 0.11 mg/dL; BUN, 57.48 ± 12.25 mg/dL; Ccr, 0.48 ± 0.06 mL/min/kg*·*bw).

### 3.2. Dehydration for 48 Hours before Iodixanol (10 mL/kg) Administration Is Optimal for Inducing CI-AKI in 5/6 NE Rats Six Weeks after the Ablative Surgery

**Figure 3:**
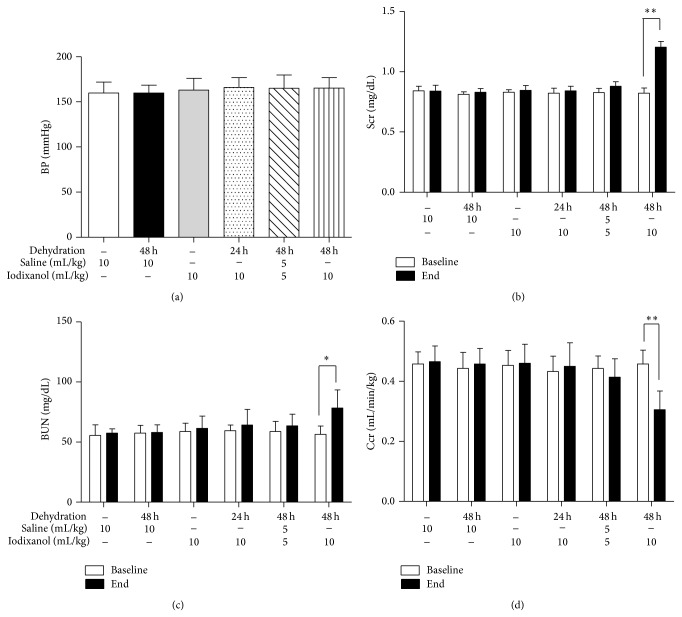
A marked reduction in renal function was induced by dehydration for 48 hours before iodixanol administration in 5/6 NE rats. (a) BP levels were not different among the six groups before saline or ICM injection. Changes in the levels of (b) Scr, (c) BUN, and (d) Ccr before and after an intravenous injection of iodixanol or saline. Animals showed marked deterioration of renal function 24 hours after iodixanol injections in the 48 h dehydration + iodixanol group. In the saline group, the 48 h dehydration + saline group, the iodixanol group, the 24 h dehydration + iodixanol group, and the 48 h dehydration + low dose iodixanol group, there were no statistical changes of renal function between baseline and final levels (Figures 2(b)–2(d)). ^**^ *P* < 0.01 and ^*^ *P* < 0.05 end point versus baseline in every group; *n* = 5.

As Figure 3 shows, baseline levels of BP, Scr, BUN, and Ccr did not differ among the groups. Compared with baseline levels, there were significant increases in Scr and BUN levels and a decrease in Ccr level 24 hours after iodixanol (10 mL/kg) injection in the 48 h dehydration + iodixanol group (Scr, 1.21 ± 0.11 versus 0.82 ± 0.09 mg/dL; BUN, 78.40 ± 14.99 mg/dL versus 56.40 ± 6.88; Ccr, 0.31 ± 0.06 versus 0.45 ± 0.05 mL/min/kg*·*bw). In the saline group, the 48 h dehydration + saline group, the iodixanol group, the 24 h dehydration + iodixanol group, and the 48 h dehydration + low dose iodixanol group, there were no statistical differences between baseline and final levels of Scr, BUN, and Ccr (Figures 3(b)–3(d)). According to definition of CI-AKI as an increase in SCr ⩾25% of the baseline value, all rats in the 48 h dehydration + iodixanol group and only one rat in the 48 h dehydration + low dose iodixanol group reached this threshold.

**Figure 4:**
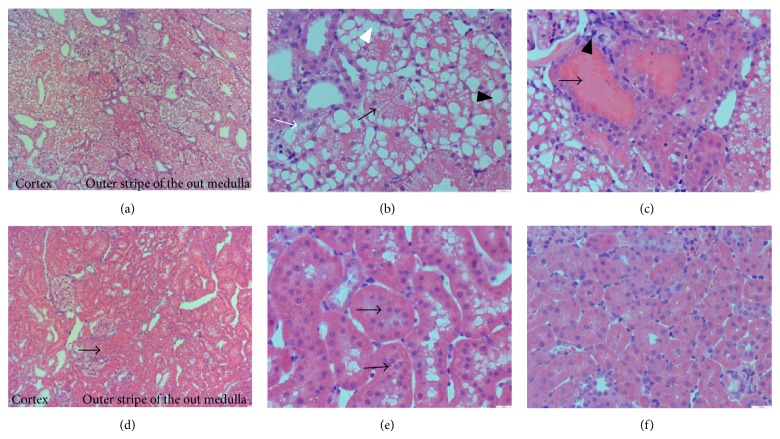
Renal morphologic injury was induced by combined dehydration for 48 hours with iodixanol (10 mL/kg) administration in 5/6 NE rats. ((a)–(c)) Representative photomicrographs of kidney injury from experimental rats treated with dehydration for 48 hours and iodixanol (10 mL/kg). (a) Representative photomicrographs of the most severe and pronounced alterations were observed in the renal corticomedullary boundary zone (the cortex and outer stripe of the outer medulla). (b) Representative photomicrographs of tubular dilation (black arrow), foamy degeneration (black arrow head), detachment of tubular cells (white arrow), and naked basement membranes (white arrow head) were observed. (c) Representative photomicrographs of proteinaceous casts (arrow) and inflammatory cell infiltration (arrow head) were observed. ((d), (e)) Representative photomicrographs of proliferation and hypertrophy (arrow) in tubular epithelial cells in 5/6 NE rats six weeks after the ablative surgery. (f) Representative photomicrographs of the renal morphology in a healthy rat. Original magnifications: ×100 ((a), (d)); ×400 ((b), (c), (e), (f)). Hematoxylin and eosin stain. Calibration bar = 20 *μ*m.

As Figures 4(a)–4(c) show, severe renal morphologic damage was observed in the renal corticomedullary boundary zone (the cortex and outer stripe of the outer medulla), including tubular dilation, detachment, naked basement membranes and foamy degeneration of tubular cells, proteinaceous or cellular casts, and inflammatory cell infiltration in the 48 h dehydration + iodixanol group. By contrast, renal morphology did not significantly differ in rats treated with the other pretreatment protocol in the saline group, the 48 h dehydration + saline group, the iohexol group, the 24 h dehydration + iohexol group, and the 48 h dehydration + low dose iodixanol group. As Figures 4(d) and 4(e) show, proliferation and hypertrophy in tubular epithelial cells was observed in 5/6 NE rats six weeks after the ablative surgery. For comparison, Figure 4(f) represents the renal morphology in a healthy rat.

### 3.3. Iohexol Resulted in Severe Kidney Damage Accompanied by More Severe Intrarenal Hypoxia and More Cellular Apoptosis, but Not Increased Inflammation and Higher Oxidative Stress Levels Compared with Iodixanol

#### 3.3.1. Iohexol Resulted in Marked Deterioration of Renal Function Compared with Iodixanol

**Figure 5:**
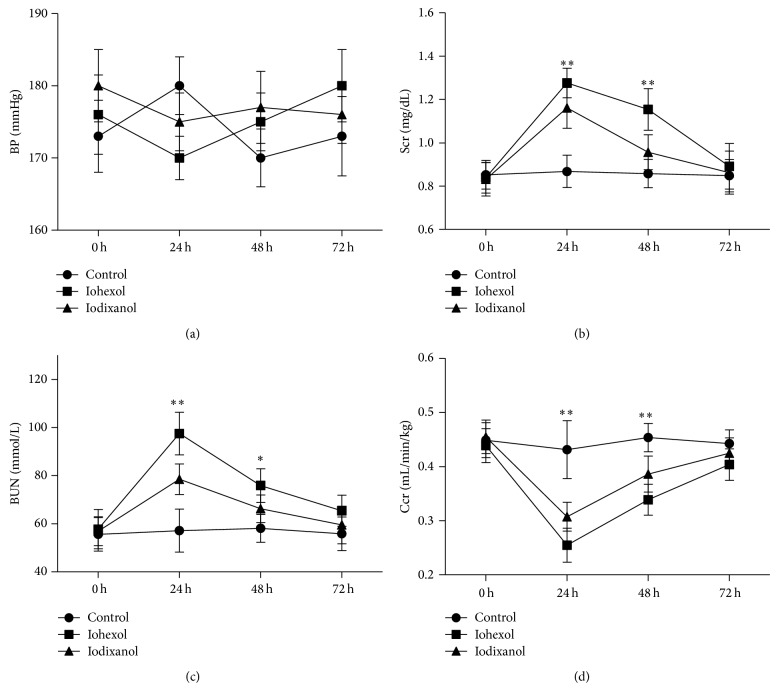
Iohexol resulted in a more significant reduction of renal function compared with iodixanol in 5/6 NE rats. Changes in the levels of (a) BP, (b) Scr, (c) BUN, and (d) Ccr before and at 24 h, 48 h, and 72 h after an intravenous injection of saline, iohexol, or iodixanol. ^**^ *P* < 0.01 and ^*^ *P* < 0.05 versus the control group; *n* = 8.

As Figure 5 shows, there were no significant differences among three groups before (0 h) and 24 h, 48 h, 72 h after injection in terms of levels of BP. In the iohexol and iodixanol groups, there were significant increases in Scr and BUN levels and a decrease in Ccr compared with the control group, which peaked at 24 h after injection. When the iohexol and iodixanol groups were compared, Scr and BUN levels were significantly higher and Ccr was markedly decreased in the iohexol group at 24 h and 48 h after injection (24 h: Scr, 1.28 ± 0.07 mg/dL versus 1.16 ± 0.09 mg/dL; BUN, 97.5 ± 8.9 mg/dL versus 78.5 ± 6.4 mg/dL; Ccr, 0.26 ± 0.03 mL/min/kg versus 0.31 ± 0.03 mL/min/kg, *P* < 0.01; 48 h: Scr, 1.15 ± 0.10 mg/dL versus 0.96 ± 0.08 mg/dL; BUN, 75.9 ± 7.0 mg/dL versus 66.3 ± 5.7 mg/dL; Ccr, 0.34 ± 0.03 mL/min/kg versus 0.39 ± 0.03 mL/min/kg, *P* < 0.01 or 0.05). There were no differences in Scr, BUN, and Ccr levels between iohexol group and iodixanol group at 72 h after injection (Scr, 0.89 ± 0.10 mg/dL versus 0.86 ± 0.10 mg/dL; BUN, 65.4 ± 6.5 mg/dL versus 59.5 ± 7.8 mg/dL; Ccr, 0.40 ± 0.03 mL/min/kg versus 0.43 ± 0.03 mL/min/kg, *P* > 0.05).

#### 3.3.2. Iohexol Resulted in More Severe Morphological Damage Compared with Iodixanol

**Figure 6:**
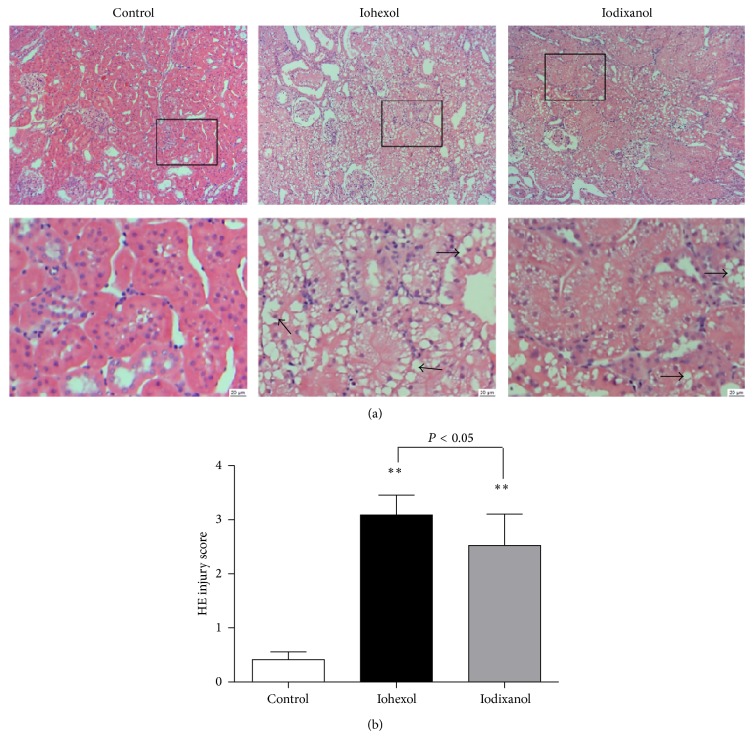
Iohexol resulted in more severe morphological injury compared with iodixanol in 5/6 NE rats. (a) Representative photomicrographs of tubular cell injury (arrow) in rat kidney tissue sections of the control, iohexol, and iodixanol groups. (b) Quantitative analysis of histologic scoring. Original magnifications: ×100. Hematoxylin and eosin stain. Calibration bar = 20 *μ*m. ^**^ *P* < 0.01 versus the control group; *n* = 8.

As Figure 6 shows, there were no noticeable detachment or foamy degeneration of tubular cells in the control group (histologic scoring: 0.41 ± 0.15), severe detachment and foamy degeneration of tubular cells in the iohexol group, and less detachment and foamy degeneration of tubular cells in the iodixanol group. Kidney injury histologic scoring of the iohexol group was higher compared with the iodixanol group (3.19 ± 0.37 versus 2.53 ± 0.58, *P* < 0.01).

#### 3.3.3. Iohexol and Iodixanol Resulted in Similarly Marked Increases in Inflammation and Oxidative Stress Levels

**Figure 7:**
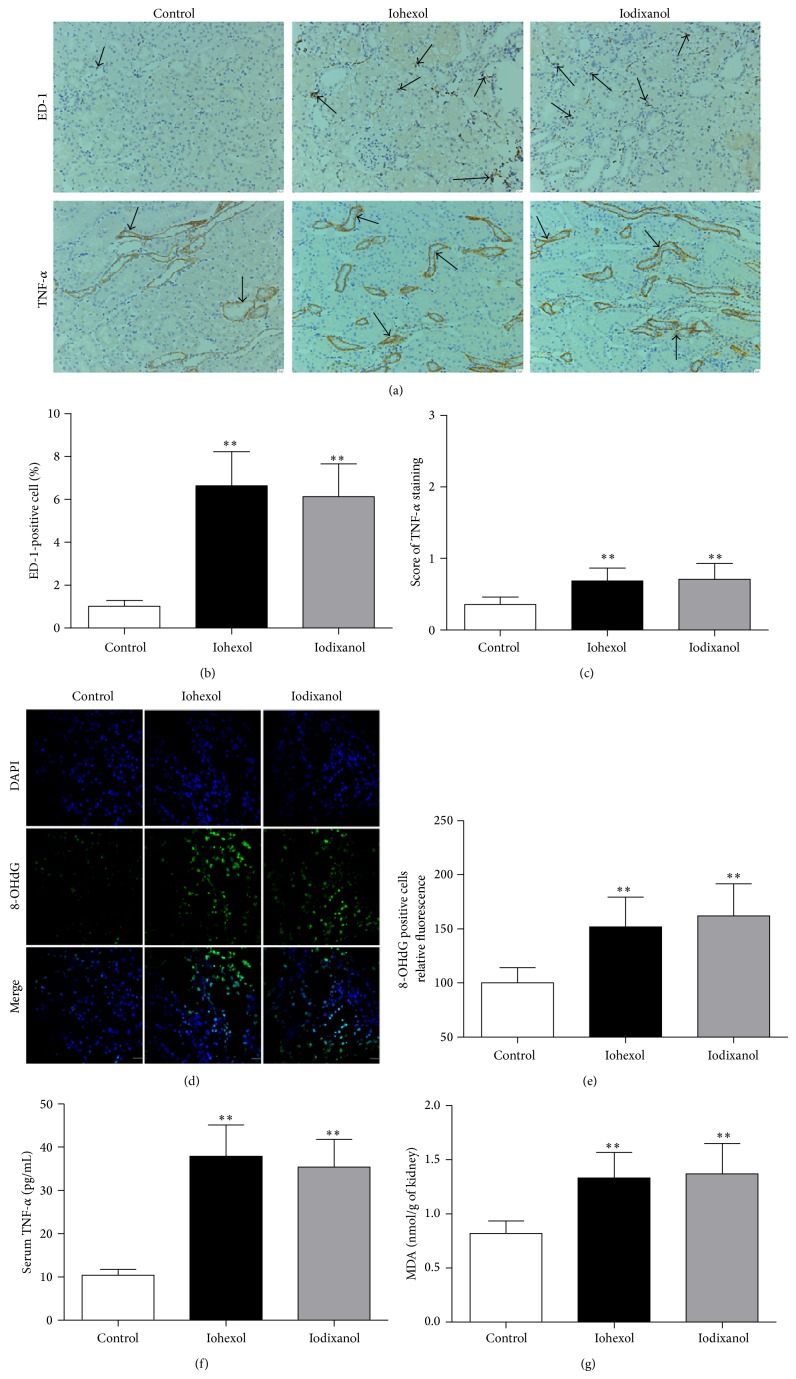
Iohexol and iodixanol resulted in similarly marked increases in inflammation and oxidative stress levels. (a) Representative photomicrographs of immunostaining for ED-1 and TNF-*α* in the renal sections of the control, iohexol, and iodixanol groups. Brown color indicates positive staining (arrow). (b) Quantitative analysis of ED-1-positive cells in the three groups by the percentage of ED-1-positive cells. (c) Quantitative analysis of the extent and intensity of TNF-*α* staining. (d) Representative photomicrographs of immunofluorescent labeling (green) for the redox product oxidized derivative of 8-OHdG in the renal sections of the control, iohexol, and iodixanol groups. Nuclei were stained with DAPI (blue). (e) Quantitative analysis of the extent and intensity of 8-OHdG-positive cells. (f) Serum TNF-*α* levels. (g) MDA concentrations in renal tissues. Original magnifications: ×200 (a); ×630 (d). Calibration bar = 20 *μ*m. ^**^ *P* < 0.01 versus the control group; *n* = 8.

To explore the effect of ICM on inflammatory responses, the protein expression of a macrophage/monocyte marker (ED-1) and tumor necrosis factor-alpha (TNF-*α*) on kidney sections and serum TNF-*α* concentrations were examined. Immunohistochemical staining revealed that the expression of ED-1-positive cells (macrophages) was abundant in peritubular spaces and TNF-*α* was mainly localized to the renal tubule epithelial cells (Figure 7(a)). Quantitatively, expression of both proteins increased significantly in the iohexol and iodixanol groups compared with the control group (*P* < 0.01). However, there were no significant differences between the iohexol and iodixanol groups in terms of the expression of both proteins (Figures 7(b) and 7(c)). In the iohexol and iodixanol groups, levels of serum TNF-*α* were markedly higher compared with the control group (*P* < 0.01). However, there were no significant differences between the iohexol and iodixanol groups in terms of serum TNF-*α* levels (Figure 7(f)).

To explore the effect of ICM on oxidative stress levels, we examined the expression of an oxidized derivative of deoxyguanosine (8-OHdG) on kidney sections and the amount of lipoperoxidation final reaction substance, that is, malondialdehyde (MDA), in the supernatant of the renal cortical homogenate. Kidney sections were subjected to immunofluorescent labeling for 8-OHdG. In the iohexol and iodixanol groups, the extent and intensity of 8-OHdG-positive cells were markedly increased compared to the control group (*P* < 0.01). However, there were no significant differences between the two groups in terms of the extent and intensity of 8-OHdG-positive cells (Figures 7(d) and 7(e)). In the iohexol and iodixanol groups, levels of MDA were markedly increased compared to the control group. However, there were no significant differences between the two groups in MDA levels (Figure 7(g)).

#### 3.3.4. Iohexol Results in Marked Diuresis and Intrarenal Hypoxia Compared with Iodixanol

**Figure 8:**
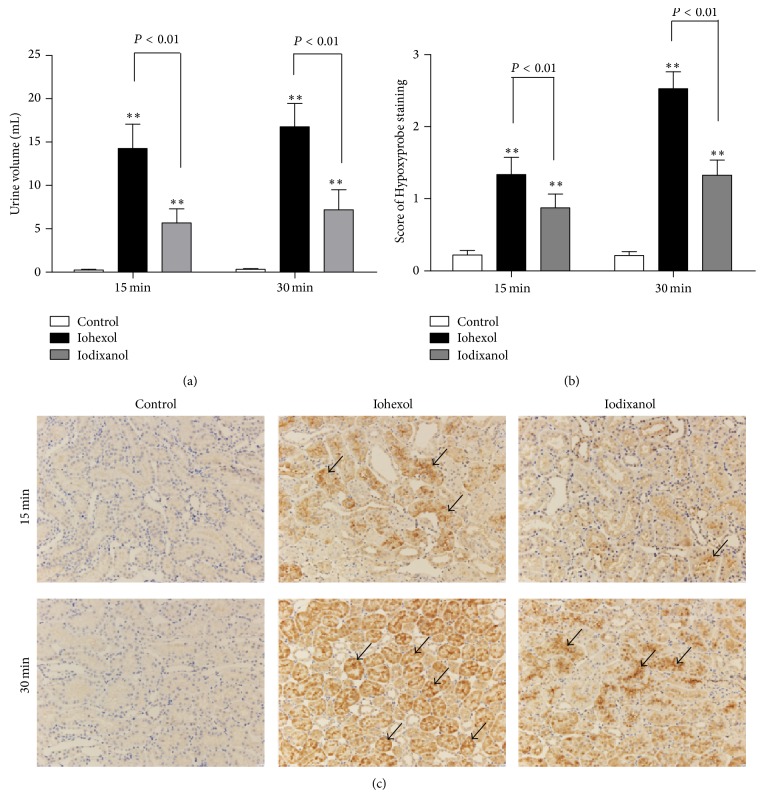
Urine volume, renal hypoxic conditions, and immunofluorescent labeling for TUNEL in the control, iohexol, and iodixanol groups. (a) Urine volume of rats in the three groups (recorded from the start of the bolus injection to 15 min or 30 min after the injection). The urine volume of rats in the iohexol group is more than that in the iodixanol group. (b) Quantitative analysis of the extent and intensity of renal Hypoxyprobe in the three groups. A little staining in the representative immunostaining of the control group, more staining in the representative immunostaining of the iohexol group compared with the control group, and less staining in the representative immunostaining of the iodixanol group. (c) Representative photomicrographs of Hypoxyprobe (anti-pimonidazole protein adducts antibody) immunostaining in renal sections 15 min and 30 min after a saline, iohexol, or iodixanol injection. Brown color indicates positive staining (arrow). Original magnifications: ×200. Calibration bar = 20 *μ*m. ^**^ *P* < 0.01 versus the control group; *n* = 8. Data are means ± SD.

The urine volume of rats was recorded from the start of the bolus injection to 15 min or 30 min after the injection. In the iohexol and iodixanol groups, the urine volume of rats was markedly increased compared with the control group over the two periods (*P* < 0.01). In the iohexol group, the urine volume of rats significantly increased compared with the iodixanol group (from the start of the bolus injection to 15 minutes after the injection: 14.25 ± 2.82 versus 5.65 ± 1.63 mL, *P* < 0.01; from the start of the bolus injection to 30 min after the injection: 16.75 ± 2.71 versus 7.20 ± 2.30 mL, *P* < 0.01) (Figure 8(a)).

Immunohistochemical staining revealed that the expression of Hypoxyprobe was mainly localized to the renal tubule epithelial cells (Figure 8(c)). In the iohexol and iodixanol groups, the expression of Hypoxyprobe 15 min and 30 min after saline, iohexol, or iodixanol injection was markedly increased compared to the control group (*P* < 0.01). Expression of Hypoxyprobe in the iohexol group was significantly higher compared with the iodixanol group (15 min and 30 min staining scoring: 1.34 ± 0.24 versus 0.87 ± 0.19 and 2.53 ± 0.23 versus 1.32 ± 0.21, resp., *P* < 0.01) (Figures 8(b) and 8(c)).

#### 3.3.5. Iohexol Results in More Apoptotic Tubular Cells Compared with Iodixanol

**Figure 9:**
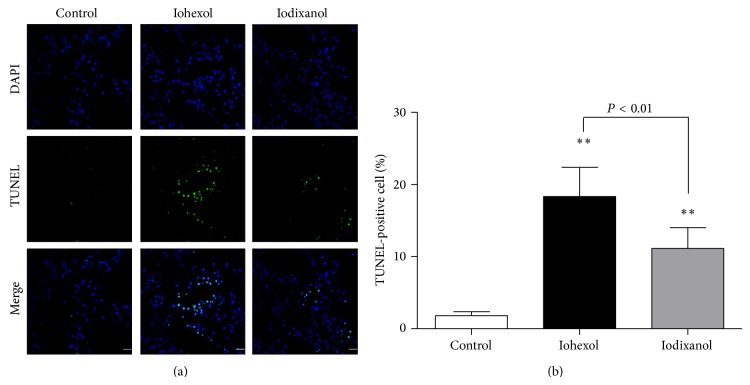
Immunofluorescent labeling for TUNEL in rat kidney tissue sections of the control, iohexol, and iodixanol groups. (a) Representative photomicrographs of TUNEL-positive cells in renal sections. Rat kidney tissue was stained for TUNEL (green). Nuclei were stained with DAPI (blue). Few TUNEL-positive cells in the representative immunofluorescent staining of the control group. A large number of TUNEL-positive cells in the representative immunofluorescent staining of iohexol group compared with control group, and less TUNEL-positive cells in the representative immunofluorescent staining of the iodixanol group. (b) Quantitative analysis of TUNEL-positive cells in the three groups by the percentage of TUNEL-positive cells. Original magnifications: ×630. Calibration bar = 20 *μ*m. ^**^ *P* < 0.01 versus the control group; *n* = 8. Data are means ± SD.

In the iohexol and iodixanol groups, apoptotic cells were markedly increased compared with the control group (*P* < 0.01). When the iohexol group was compared with the iodixanol group, the number of apoptotic cells was significantly higher in the iohexol group (percentage of TUNEL-positive cells: 18.3 ± 4.1 versus 11.2 ± 2.9, *P* < 0.01) (Figures 9(a) and 9(b)).

## 4. Discussion

Animal models are needed to better understand the pathogenic mechanisms of CI-AKI that will help to design new therapeutic approaches for speeding up patient recovery [17]. The 5/6 NE rat model is widely used to study CKD. Several models are based on the 5/6 NE rat [18–20]. In the present study, we successfully established a new CI-AKI model that is based on the 5/6 NE rat at the optimal time after the kidney ablative surgery.

Renal insufficiency and hypertension are involved in the susceptibility to CI-AKI in the 5/6 NE rat. However, the resistance to contrast-induced nephrotoxicity in healthy animals is significantly high [21]. Simple intensification of a single insult does not lead to reliable models. Therefore pretreating rats with additional insults is essential to establish a CI-AKI rat model.

Predisposing factors should be similar to human risk factors. It is the responsibility of researchers to approach it as closely as possible. In previous studies, pretreatment included introducing ischemic damage to the kidneys [2], some nephrotoxic drugs (e.g., aminoglycoside, cyclosporine A, glycerol, cisplatin, Adriamycin, etc.) [5, 6, 16], and dehydration [8, 10]. The nephrotoxic drug and ischemic damage are confounding factors and interfere with results in research [2]. However, water deprivation can be prepared with no pharmacological procedures, and Efrati et al. [8] demonstrated that manifestations of CM-induced renal vasoconstriction and oxidative stress are the most prominent in a dehydrated animal. Thus, water deprivation is considered an appropriate insult and is advantageous to study the underlying mechanism of CI-AKI.

The result shows that only dehydration for 48 h does not result in deterioration of renal function in the 5/6 NE rat. Depriving rats of water for 48 h hardly influences their blood volumes or results in their hypovolemia. Moreover, depriving animals of water can decrease amount of physical food and activity elevating BUN and Cr levels. Therefore levels of Scr, BUN, and Ccr in rats without ICM injection underwent the dehydration for 48 h were not changed (Figure 2).

These morphological findings in our study closely resemble the findings in kidney biopsies from patients with CI-AKI [22]. The locations of renal histopathological changes were similar to those reported in the rats and clinically in patients whom the proximal tubules were mostly affected [17, 22].

The iohexol group had severe tubular injury whereas the iodixanol group had moderate to severe tubular injury. The pathogenesis of CI-AKI is conceivably a paradigm of hypoxic/toxic injury, involving altered renal microcirculation, hypoxia, and reactive oxygen species-mediated cellular injury [2]. The iohexol group had significantly more intrarenal hypoxia than the iodixanol group. The number of apoptotic cells was also significantly higher in the iohexol group than in the iodixanol group. However, inflammation and oxidative stress levels between the two groups were similar. These results indicate that iohexol caused increased nephrotoxicity compared with iodixanol because of more intrarenal hypoxia and more apoptosis.

Dimeric iodine contrast agents have high viscosity and cause prolonged iodine retention and low perfusion in kidney [23, 24]. The increased urine viscosity by iodixanol directly decreases the glomerular filtration rate due to the high pressure within the tubule lumen and the low net driving force for glomerular filtration. When there is more reabsorption of filtrate because of volume depletion, the viscosity in the urine would be greater. Therefore there was intrarenal hypoxia in the iodixanol group. Iohexol has high viscosity and osmolality among the LOCMs. Viscosity and osmolality both contribute to nephrotoxicity. Iohexol decreases extracellular volume contraction. The direct vasoconstrictor effects of iohexol and further exacerbation of ischemia are significant because the vasoconstrictor hormones (e.g., rennin, endothelin, and adenosine) increase and the vasodilator hormones (e.g., prostaglandin and nitric oxide) decrease. In the present study, the experimental rats were already volume depleted and showed more urine output than the iodixanol group (Figure 7(a)) and extracellular volume depletion would be exacerbated resulting in a greater stimulation of intrarenal vasoconstrictor factors [25]. Thus iohexol resulted insignificantly in more intrarenal hypoxia than iodixanol.

However, ICM was previously shown to increase or decrease renal medullary blood flux in different studies [26–28]. Seeliger et al. [24] reported that iodixanol induced less renal medullary blood flux than iohexol. Taken together, the experimental setting seems to be highly important for determining the final effect of ICM on renal medullary blood flux.

The extent of kidney injury induced by the same iodinated dose iohexol and iodixanol was different. Jensen et al. [29] reported that LOCM induced more markedly direct toxic effect on tubular epithelial cells than IOCM. This is consistent with our result. Other clinical studies have shown similar results. The NEPHRIC trial showed that the incidence of CI-AKI with iodixanol was significantly lower than with iohexol in this high-risk population [30]. A meta-analysis showed that the incidence of CI-AKI induced by iohexol was higher than that caused by IOCM [31]. However, Barrett et al. reported no benefit of the dimeric isoosmolar ICM versus another monomeric, less viscous ICM in a study of 153 patients [32].

## 5. Conclusions

The rat with 5/6 NE, a model of ablative nephropathy, is a reliable and suitable small animal model for LOCM- or IOCM-induced AKI similar to clinical CI-AKI. We found that nephrotoxicity induced by iohexol was significantly more than that caused by iodixanol* in vivo*. Iohexol induced more severe AKI than iodixanol due to increased intrarenal hypoxia and apoptotic tubular cells. The different osmolalities of the contrast media were shown to correlate with several different indices of cellular toxicity and support the conclusion that this is a major contributory factor in CI-AKI.
